# (*N*,*N*-Diiso­propyl­dithio­carbamato)tri­phenyl­tin(IV): crystal structure, Hirshfeld surface analysis and computational study

**DOI:** 10.1107/S2056989019012490

**Published:** 2019-09-12

**Authors:** Farah Natasha Haezam, Normah Awang, Nurul Farahana Kamaludin, Mukesh M. Jotani, Edward R. T. Tiekink

**Affiliations:** a Environmental Health and Industrial Safety Programme, Faculty of Health Sciences, Universiti Kebangsaan Malaysia, Jalan Raja Muda Abdul Aziz, 50300 Kuala Lumpur, Malaysia; b Department of Physics, Bhavan’s Sheth R. A. College of Science, Ahmedabad, Gujarat 380001, India; c Research Centre for Crystalline Materials, School of Science and Technology, Sunway University, 47500 Bandar Sunway, Selangor Darul Ehsan, Malaysia

**Keywords:** crystal structure, organotin, di­thio­carbamate, Hirshfeld surface analysis, computational chemistry

## Abstract

The crystal and mol­ecular structures of the title triorganotin di­thio­carbamate, [Sn(C_6_H_5_)_3_(C_7_H_14_NS_2_)], are described. The mol­ecular geometry about the metal atom is highly distorted being based on a C_3_S tetra­hedron as the di­thio­carbamate ligand is asymmetrically chelating to the tin centre. The close approach of the second thione-S atom [Sn⋯S = 2.9264 (4) Å] is largely responsible for the distortion. The mol­ecular packing is almost devoid of directional inter­actions with only weak phenyl-C—H⋯C(phen­yl) inter­actions, leading to centrosymmetric dimeric aggregates, being noted. An analysis of the calculated Hirshfeld surface points to the significance of H⋯H contacts, which contribute 66.6% of all contacts to the surface, with C⋯H/H⋯C [26.8%] and S⋯H/H⋯H [6.6%] contacts making up the balance.

## Abstract 2

The tin coordination geometry in (C_6_H_5_)_3_Sn[S_2_CN(*i*-Pr)_2_] is based on a tetra­hedron but the geometry of the C_3_S donor set is distorted by the close proximity of the second thione-S atom. In the crystal, weak C—H⋯C inter­actions link mol­ecules into centrosymmetric dimers.

## Chemical context   

Organotin(IV) compounds have long been investigated as potential anti-cancer agents (Gielen & Tiekink, 2005 ▸) and studies in this area continue. Further, organotin compounds have received much attention owing to their potential therapeutic potential as anti-fungal, anti-bacterial, anti-malarial and schizonticidal agents (Khan *et al.*, 2014 ▸). Metal di­thio­carbamates have also encouraged much inter­est in the context of chemotherapeutic agents (Hogarth, 2012 ▸) and these include organotin(IV) di­thio­carbamate compounds (Tiekink, 2008 ▸; Adeyemi & Onwudiwe, 2018 ▸). In view of the wide-range of applications/potential of organotin(IV) di­thio­carbamate compounds and in continuation of on-going studies in this area (Khan *et al.*, 2015 ▸; Mohamad *et al.* 2016 ▸, 2017 ▸, 2018 ▸), the title compound, Ph_3_Sn[S_2_CN(*i*-Pr)_2_], (I), was synthesized and characterized spectroscopically. Herein, the crystal and mol­ecular structures of (I) are described along with a detailed analysis of the mol­ecular packing *via* the calculated Hirshfeld surfaces and computational chemistry study.
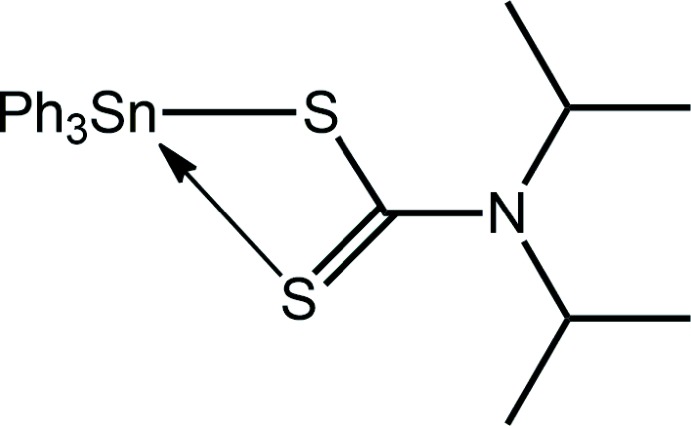


## Structural commentary   

**Figure 1:**
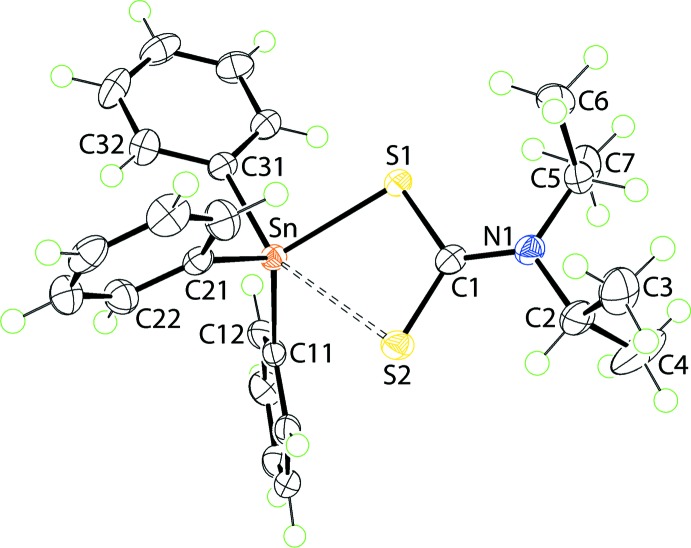
The mol­ecular structure of (I) showing the atom-labelling scheme and displacement ellipsoids at the 70% probability level. The long Sn1⋯S2 contact is indicated by a double-dashed line.

The tin atom in (I), Fig. 1 ▸, is coordinated by an asymmetrically bound di­thio­carbamate ligand and three *ipso*-carbon atoms of the phenyl groups (Table 1 ▸). The disparity in the Sn—S separations, *i.e*. Δ(Sn—S) = [(Sn—S_l_) − (Sn—S_s_)] = 0.45 Å (l = long, s = short), is rather great suggesting that the Sn⋯S2 inter­action is weak. This is supported in the pattern of C—S bond lengths with that involving the more tightly bound S1 atom being nearly 0.06 Å longer than the equivalent bond with the weakly bound S2 atom. Despite this, a clear influence of the S2 atom is noted on the Sn—C bond lengths with the Sn—C31 bond length being significantly longer than the other Sn—C bonds, Table 1 ▸. The S2—Sn—C31 bond angle is 158.41 (4)° and is suggestive of a *trans*-influence exerted by the S2 atom; there is no other *trans* angle about the tin atom. If the coordination geometry is considered as tetra­hedral, the range of tetra­hedral angles is 93.24 (4)°, for S1—Sn—C31, to 119.87 (6)°, for C11—Sn—C21. The range of angles assuming a five-coordinate, C_3_S_2_, geometry is 65.260 (11)°, for the S1—Sn—S2 chelate angle to the aforementioned 158.41 (4)°. A descriptor for assigning coordination geometries to five-coordinate species is τ (Addison *et al.*, 1984 ▸). In the case of (I), this computes to 0.64, which indicates a geometry somewhat closer to an ideal trigonal bipyramid (τ = 1.0) than to an ideal square pyramid (τ = 0.0). Also included in Table 1 ▸ are the C—N bond lengths, which show C1—N1 to be significantly shorter than the C2—N1 and C5—N1 bond lengths, an observation consistent with a significant contribution of the ^2−^S_2_C=N^+^(*i*-Pr)_2_ canonical form to the overall electronic structure of the di­thio­carbamate ligand.

## Supra­molecular features   

**Figure 2:**
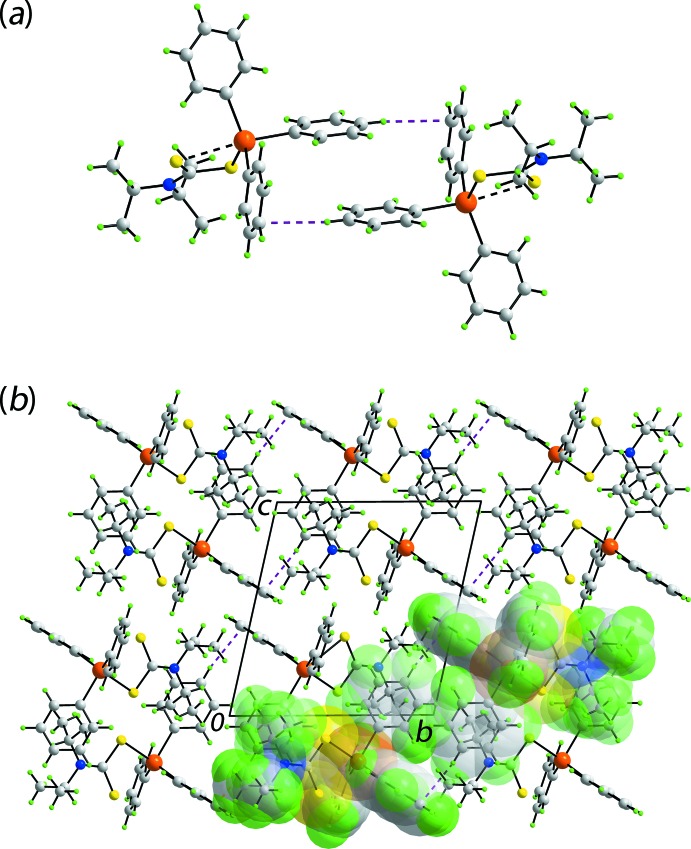
Mol­ecular packing in the crystal of (I): (*a*) supra­molecular dimer sustained by localized phenyl-C—H⋯C(phen­yl) inter­actions shown as purple dashed lines and (*b*) a view of the unit-cell contents in projection down the *a* axis. One column of dimeric aggregates is highlighted in space-filling mode.

The geometric parameters characterizing the identified inter­molecular inter­action operating in the crystal of (I) are collated in Table 2 ▸. A phenyl-C—H⋯C(phen­yl) contact less than the sum of the sum of the Waals radii (Spek, 2009 ▸) is noted to occur between centrosymmetrically related mol­ecules, Fig. 2 ▸(*a*). This is an example of a localized C—H⋯π contact whereby the hydrogen atom directed towards a single carbon atom of the ring as opposed to a delocalized inter­action where the hydrogen atom (or halide atom or lone-pair of electrons) is directed towards the centroid of the ring (Schollmeyer *et al.*, 2008 ▸; Tiekink, 2017 ▸).

## Hirshfeld surface analysis   

**Figure 3:**
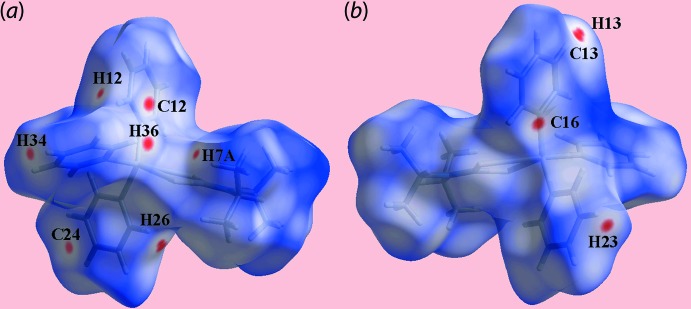
Two views of Hirshfeld surface for (I) mapped over *d*_norm_ in the range −0.085 to +1.355 (arbitrary units), highlighting short inter­atomic H⋯H and C⋯H/H⋯C contacts as diminutive red spots near the respective atoms.

**Figure 4:**
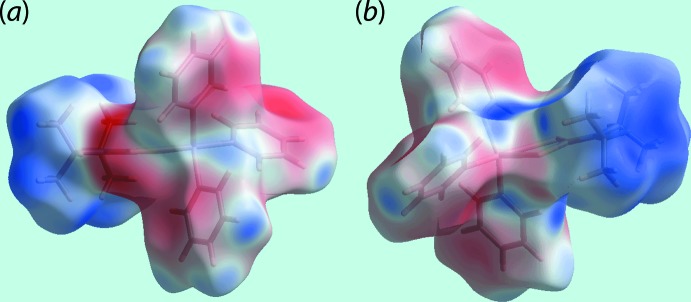
Two views of Hirshfeld surface mapped over the electrostatic potential (the red and blue regions represent negative and positive electrostatic potentials, respectively) in the range −0.032 to +0.035 atomic units.

The Hirshfeld surface calculations for (I) were performed employing *Crystal Explorer 17* (Turner *et al.*, 2017 ▸) and recently published protocols (Tan *et al.*, 2019 ▸). In the absence of classical hydrogen bonds, the influence of the localized C—H⋯π inter­action, Table 2 ▸, as well as inter­atomic H⋯H and C⋯H/H⋯C contacts, Table 3 ▸, upon the mol­ecular packing are evident as the diminutive-red spots near the participating carbon and hydrogen atoms on the Hirshfeld surfaces mapped over *d*_norm_ in Fig. 3 ▸. It is also noted that with the exception of the methyl-H7*A* atom, all of the specified inter­atomic contacts only involve the carbon and hydrogen atoms of the coordin­ated phenyl rings, Table 3 ▸. On the Hirshfeld surface mapped over electrostatic potential in Fig. 4 ▸, the light-blue and faint-red regions, corresponding to positive and negative electrostatic potential, respectively, occur about the di-*iso*-propyl and tri­phenyl­tin groups, respectively.

**Figure 5:**
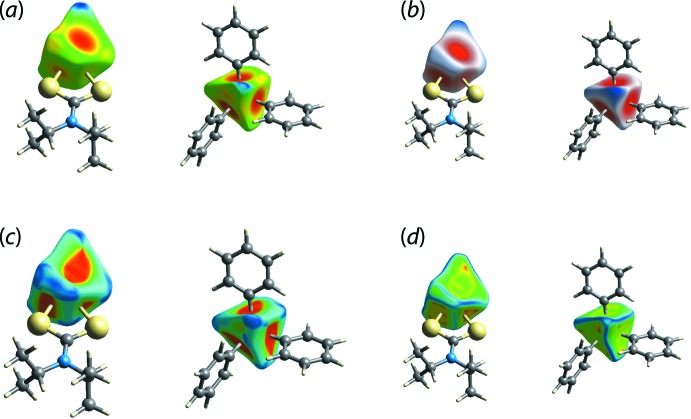
The Hirshfeld surfaces of the tin centre in (I) highlighting the coordination by the di­thio­carbamate ligand (left-hand images) and the phenyl rings (right-hand images) mapped over (*a*) the distance, *d*_e_, external to the surface in the range −0.981 to 2.436 Å, (*b*) *d*_norm_ in the range −0.890 to +1.135 Å, (*c*) the shape-index (S) from −1.0 to +1.0 (arbitrary units) and (*d*) curvedness (C) from −4.0 to +0.4 (arbitrary units).

**Figure 6:**
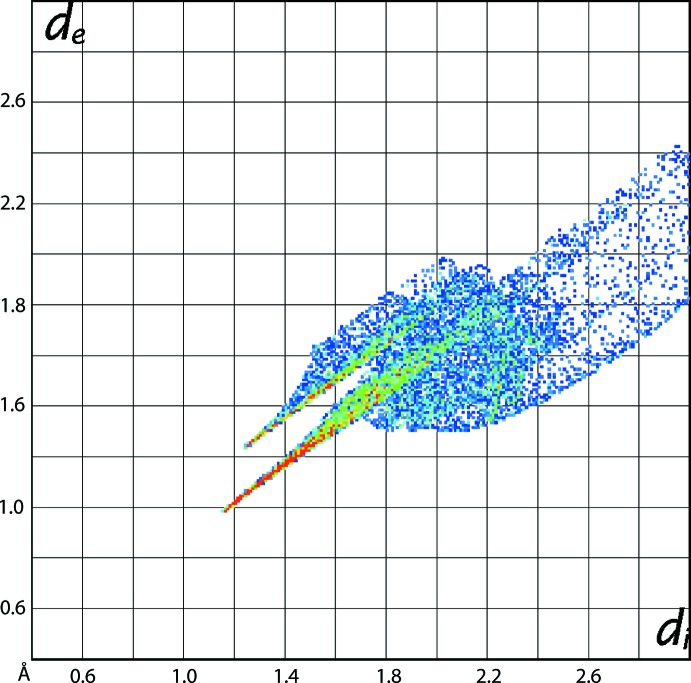
The fingerprint plot taking into account only the Hirshfeld surface about the tin atom.

As reported recently (Pinto *et al.*, 2019 ▸), in addition to analysing the nature and strength of inter­molecular inter­actions among mol­ecules, the analyses of Hirshfeld surfaces can also provide useful insight into metal–ligand/donor atom inter­actions in coordination compounds. Thus, the distance from the surface to the nearest external (*d*_e_) and inter­nal (*d*_i_) nuclei, the shape-index (*S*) and the curvedness (*C*) can also be plotted. Accordingly, Fig. 5 ▸ illustrates the Hirshfeld surfaces for the tin atom coordinated by di­thio­carbamate ligand as well as by the three phenyl groups. The close proximity of the di­thio­carbamate-S1 and phenyl-C11, C21 and C31 atoms to the tin centre are characterized as bright-red regions perpendicular to bond directions on the Hirshfeld surfaces mapped over *d*_e_, Fig. 5 ▸(*a*), and *d*_norm_, Fig. 5 ▸(*b*), whereas the comparatively weak Sn—S2 inter­action appears as the faint-red region. The longer Sn—C31 bond compared to other two Sn—C bonds, Table 1 ▸, is also characterized from these Hirshfeld surfaces through the curvature of the red region. The Sn—S1 and Sn—C bonds result in the large red regions on the shape-index mapping in Fig. 5 ▸(*c*) compared to a small red region for the Sn—S2 bond. On the Hirshfeld surfaces mapped over curvedness in Fig. 5 ▸(*d*), the strength of the tin–ligand bonds are characterized as the yellow areas separated by green regions. The coordination bonds for tin are also rationalized in the fingerprint plot taking into account only the Hirshfeld surface about the metal atom, Fig. 6 ▸. The distribution of green points having upper short spike at *d*_e_ + *d*_i_ ∼2.4 Å and the lower, long red spike at *d*_e_ + *d*_i_ ∼2.1 Å are the result of the Sn—S and Sn—C bonds, respectively. This asymmetric distribution of points about the diagonal lacking homogeneity in colouration is due to the distorted coordination geometry about the tin atom.

**Figure 7:**
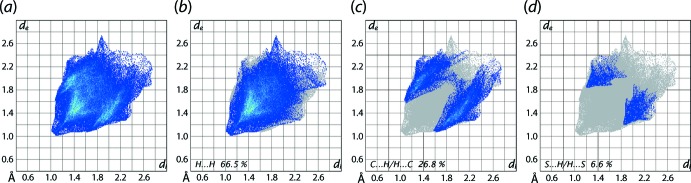
(*a*) A comparison of the full two-dimensional fingerprint plot for (I) and those delineated into (*b*) H⋯H, (*c*) C⋯H/H⋯C and (*d*) S⋯H/H⋯S contacts.

It is clear from the the calculation of the overall two-dimensional fingerprint plot for (I), Fig. 7 ▸(*a*), that the plot is asymmetric about the (*d*_e_, *d*_i_) diagonal in the longer distance regions and have contributions only from the inter­atomic contacts involving carbon, hydrogen and sulfur atoms, Table 4 ▸. The two-dimensional fingerprint plots delineated into H⋯H, C⋯H/H⋯C, C⋯C and S⋯H/H⋯S contacts are shown in Fig. 7 ▸(*b*)–(*d*), respectively. In the fingerprint plot delineated into H⋯H contacts in Fig. 7 ▸(*b*), the presence of the short inter­atomic H⋯H inter­action involving the methyl-H7*A* and phenyl-H12 atoms is evident as the pair of short overlapping peaks at *d*_e_ + *d*_i_ ∼2.1 Å with the other short inter­atomic H⋯H contact (Table 3 ▸) merged within the plot. The inter­molecular C—H⋯C inter­actions describing the localized C—H⋯π contacts are evidenced by a pronounced pair of characteristic wings around (*d*_e_, *d*_i_) ∼(1.2 Å, 1.8Å) and ∼(1.8 Å, 1.2 Å) in the fingerprint plot delineated into C⋯H/H⋯C contacts shown in Fig. 7 ▸(*c*). The other short inter­atomic C⋯H contacts summarized in Table 3 ▸ appear as the pair of forceps-like tips at *d*_e_ + *d*_i_ ∼2.7 Å. The fingerprint plot delineated into S⋯H/H⋯S contacts in Fig. 7 ▸(*d*) indicate that sulfur atoms are nearly at van der Waals separation from the symmetry-related hydrogen atoms.

## Computational chemistry   

The pairwise inter­action energies between the mol­ecules within the crystal were calculated using *Crystal Explorer 17* (Turner *et al.*, 2017 ▸) and summing up the four energy components: electrostatic (*E*_ele_), polarization (*E*_pol_), dispersion (*E*_dis_) and exchange-repulsion (*E*_rep_). The energies were obtained using the wave function calculated at the HF/STO-3G level of theory. The strength and the nature of inter­molecular inter­actions in terms of their energies are summarized in Table 5 ▸. An analysis of these reveals that the dispersion energy component makes the greatest contribution to the energies in the absence of classical hydrogen (electrostatic) bonds. Among the short inter­atomic contacts listed in Table 5 ▸, the inter­molecular phenyl-C—H36⋯C12 contact combined with the methyl-H7*A*⋯H12(phen­yl) inter­action, occurring between the same pair of symmetry-related mol­ecules, gives rise to the maximum total energy of inter­action, compared to the other inter­actions, which have almost the same energy values.

**Figure 8:**
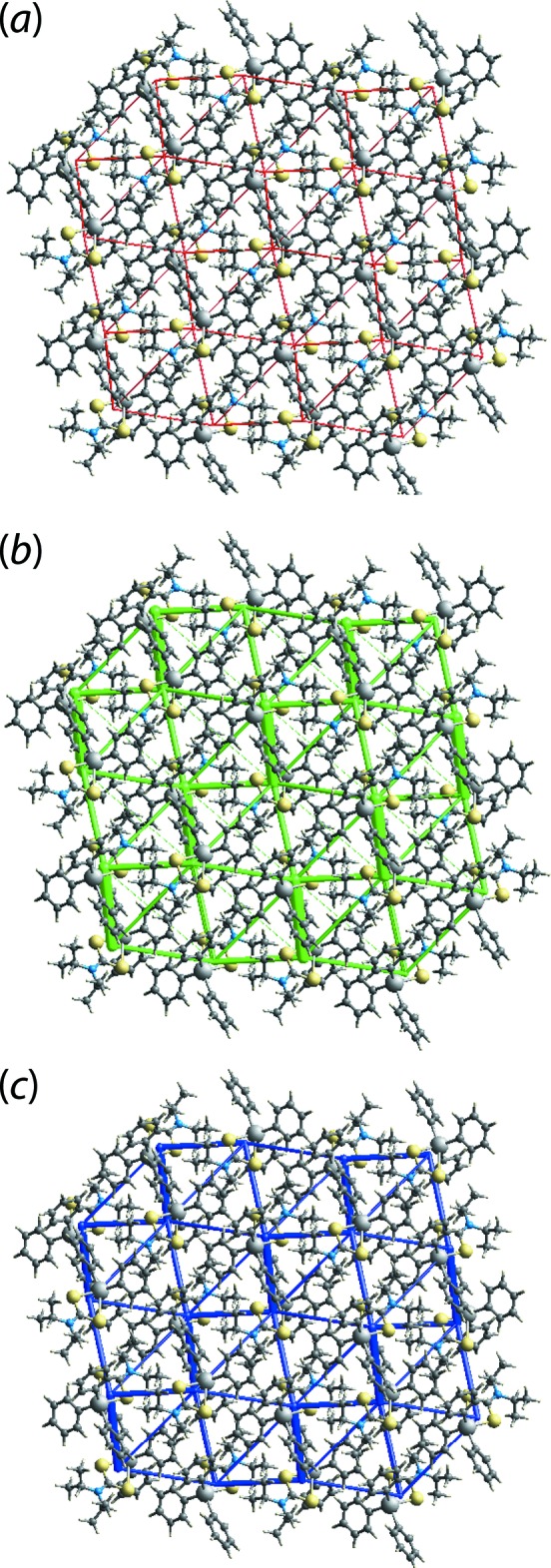
The energy frameworks calculated for (I) viewed down the *c*-axis direction showing the (*a*) electrostatic potential force, (*b*) dispersion force and (*c*) total energy. The energy frameworks were adjusted to the same scale factor of 30 with a cut-off value of 3 kJ mol^−1^ within 2 × 2 × 2 unit cells.

The graphical representation of the magnitudes of inter­molecular energies in Fig. 8 ▸, *i.e.* energy frameworks, relies on a red, green and blue colour code scheme, reflecting the *E*_ele_, *E*_disp_ and *E*_tot_ components, respectively. For the direct comparison of magnitudes of inter­action energies, their magnitudes were adjusted to same scale factor of 30 with a cut-off value of 3 kJ mol^−1^ within 2 × 2 × 2 unit cells. It is clear from Fig. 8 ▸ that the green cylinders joining the centroids of mol­ecular pairs highlighting the dispersion components make a significant contribution to the supra­molecular architecture in the crystal.

## Database survey   

As indicated in a recent report (Mohamad *et al.*, 2018 ▸), there are nearly 50 crystal structures available for mol­ecules of the general formula Ph_3_Sn(S_2_CN*RR*′) and, with a number of these having multiple mol­ecules in the asymmetric unit, there are almost 60 independent mol­ecules. These conform to the same structural motif. An analysis of the key geometric parameters defining the mode of coordination of the di­thio­carbamate ligands showed that there were no systematic variations that could be correlated with the nature of the di­thio­carbamate ligand, *i.e. R*/*R*′ substituents. This observation is borne out by DFT calculations on different organotin systems that proved the influence of mol­ecular packing on (non-systematic) geometric parameters, including metal–sulfur/halide bonds (Buntine *et al.*, 1998*a* ▸,*b* ▸, 1999 ▸). In terms of Ph_3_Sn(S_2_CN*RR*′), the mean Sn—S_s_ bond length is 2.47 Å (standard deviation = 0.013 Å) and the average Sn—S_l_ bond length is 3.04 Å (0.070 Å). The Sn—S_s_ and Sn—S_l_ bond lengths in (I) both fall within 2σ of their respective means.

The homogeneity in the mol­ecular structures of Ph_3_Sn(S_2_CN*RR*′) is quite remarkable. Structural diversity is well-established for the organotin di­thio­carbamates (Tiekink, 2008 ▸; Muthalib *et al.*, 2014 ▸) such as for mol­ecules of the general formula *R*′′_2_Sn(S_2_CN*RR*′)_2_, for which four distinct structural motifs are known (Zaldi *et al.*, 2017 ▸). Also, the behaviour of tri­phenyl­tin di­thio­carbamates contrasts the analogous chemistry of tri­phenyl­tin carboxyl­ates (Tiekink, 1991 ▸). These are often monomeric (*e.g*. Basu Baul *et al.*, 2001 ▸), as for (I), but, polymeric examples are known (*e.g*. Willem *et al.*, 1997 ▸; Smyth & Tiekink, 2000 ▸). The polymeric structures occur when the carboxyl­ate ligands are bidentate bridging, leading to *trans*-C_3_O_2_ trigonal–bipyramidal coordination geometries for the tin atoms. This fundamental difference in structural chemistry arises as a result of the significant contribution of the ^2−^S_2_C=N^+^ *RR*′ canonical form to the electronic structure of the di­thio­carbamate anion, as discussed above. The formal negative charge on each sulfur atom makes this ligand a very efficient chelator which effectively reduces the Lewis acidity of the tin centre. Far from being a curiosity, such behaviour, *i.e*. di­thio­carbamate ligands reducing the Lewis acidity of metal centres, when compared to related xanthate (^−^S_2_CO*R*) and di­thio­phopshate [(^−^S_2_P(O*R*)_2_] ligands, leads to stark differences in coordination propensities in zinc-triad element 1,1-di­thiol­ate compounds, as has been reviewed recently (Tiekink, 2018*a* ▸,*b* ▸).

## Synthesis and crystallization   

All chemicals and solvents were used as purchased without purification. The melting point was determined using an automated melting point apparatus (MPA 120 EZ-Melt). Carbon, hydrogen, nitro­gen and sulfur analyses were performed on a Leco CHNS-932 Elemental Analyzer.

Di-*is*o-propyl­amine (Aldrich; 1.41 ml, 10 mmol) dissolved in ethanol (30 ml) was stirred under ice-bath conditions at 277 K for 20 mins. A 25% ammonia solution (1–2 ml) was added to provide basic conditions. Then, a cold ethano­lic solution of carbon di­sulfide (0.60 ml, 10 mmol) was added dropwise into the solution followed by stirring for 2 h. After that, tri­phenyl­tin(IV) chloride (Merck; 3.85 g, 10 mmol) dissolved in ethanol (20–30 ml) was added dropwise into the solution followed by further stirring for 2 h. The precipitate that formed was filtered and washed a few times with cold ethanol to remove impurities. Finally, the colourless precipitate was dried in a desiccator. Recrystallization was carried out by dissolving the compound in a chloro­form and ethanol mixture (1:1 *v*/*v*). This solution was allowed to slowly evaporate at room temperature yielding colourless slabs of (I). Yield: 47%, m. p.: 437.8–440.2 K. Elemental analysis: Calculated (%): C 57.07, H 5.51, N 2.66, S 12.19. Found (%): C 57.39, H 5.31, N 2.48, S 11.26.

## Refinement   

Crystal data, data collection and structure refinement details are summarized in Table 6 ▸. Carbon-bound H atoms were placed in calculated positions (C—H = 0.95–1.00 Å) and were included in the refinement in the riding model approximation, with *U*_iso_(H) set to 1.2–1.5*U*_eq_(C).

## Supplementary Material

Crystal structure: contains datablock(s) I, global. DOI: 10.1107/S2056989019012490/hb7851sup1.cif

Structure factors: contains datablock(s) I. DOI: 10.1107/S2056989019012490/hb7851Isup2.hkl

CCDC reference: 1952056

Additional supporting information:  crystallographic information; 3D view; checkCIF report

## Figures and Tables

**Table 1:** Selected bond lengths (Å)

Sn—S1	2.4792 (4)	C1—S1	1.7587 (15)
Sn—S2	2.9264 (4)	C1—S2	1.7006 (16)
Sn—C11	2.1446 (14)	C1—N1	1.336 (2)
Sn—C21	2.1349 (15)	C5—N1	1.495 (2)
Sn—C31	2.1754 (15)	C2—N1	1.497 (2)

**Table 2:** Hydrogen-bond geometry (Å, °)

*D*—H⋯*A*	*D*—H	H⋯*A*	*D*⋯*A*	*D*—H⋯*A*
C34—H34⋯C24^i^	0.95	2.82	3.757 (3)	171

Symmetry code: (i) 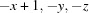.

**Table 3:** Summary of short inter­atomic contacts (Å) in (I) The inter­atomic distances are calculated in *Crystal Explorer 17* (Turner *et al.*, 2017 ▸) whereby the *X*—H bond lengths are adjusted to their neutron values.

Contact	Distance	Symmetry operation
H7*A*⋯H12	2.09	1 − *x*, 1 − *y*, −*z*
H13⋯H26	2.26	1 + *x*, *y*, *z*
C12⋯H36	2.65	1 − *x*, 1 − *y*, −*z*
C13⋯H26	2.68	1 + *x*, *y*, *z*
C16⋯H23	2.65	1 − *x*, −*y*, 1 − *z*
C24⋯H34	2.68	1 − *x*, −*y* − *z*

**Table 4:** Percentage contributions of inter­atomic contacts to the Hirshfeld surface for (I)

Contact	Percentage contribution
H⋯H	66.5
C⋯H/H⋯C	26.8
S⋯H/H⋯S	6.6
C⋯S/S⋯C	0.1

**Table 5:** Summary of inter­action energies (kJ mol^−1^) calculated for (I)

Contact	*R* (Å)	*E*_ele_	*E*_pol_	*E*_dis_	*E*_rep_	*E*_tot_
H7*A*⋯H12^i^ +	6.30					
H36⋯C12^i^		−17.5	−7.0	−112.7	68.0	−68.8
H13⋯H26^ii^ +	9.76					
C13⋯H26^ii^		−8.7	−2.0	−31.6	18.0	−24.0
C16⋯H23^iii^	9.46	−12.9	−2.0	−41.9	29.5	−28.2
C24⋯H34^iv^	10.62	−11.0	−2.8	−33.8	21.6	−26.0

Notes: Symmetry operations: (i) 1 − *x*, 1 − *y*, −*z*; (ii) 1 + *x*, *y*, *z*; (iii) 1 − *x*, −*y*, 1 − *z*; (iv) 1 − *x*, −*y*, −*z*.

**Table 6:** Experimental details

Crystal data	
Chemical formula	[Sn(C_6_H_5_)_3_(C_7_H_14_NS_2_)]
*M*_r_	526.30
Crystal system, space group	Triclinic, *P* 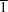
Temperature (K)	100
*a*, *b*, *c* (Å)	9.7572 (1), 11.7030 (2), 11.7602 (2)
α, β, γ (°)	74.419 (1), 80.114 (1), 67.285 (2)
*V* (Å^3^)	1189.71 (4)
*Z*	2
Radiation type	Cu *K*α
μ (mm^−1^)	10.25
Crystal size (mm)	0.12 × 0.11 × 0.08

Data collection	
Diffractometer	Rigaku XtaLAB Synergy Dualflex AtlasS2
Absorption correction	Gaussian (*CrysAlis PRO*; Rigaku OD, 2018 ▸)
*T*_min_, *T*_max_	0.840, 1.000
No. of measured, independent and observed [*I* > 2σ(*I*)] reflections	28219, 4248, 4231
*R*_int_	0.022
(sin θ/λ)_max_ (Å^−1^)	0.597

Refinement	
*R*[*F* ^2^ > 2σ(*F* ^2^)], *wR*(*F* ^2^), *S*	0.015, 0.040, 1.00
No. of reflections	4248
No. of parameters	266
H-atom treatment	H-atom parameters constrained
Δρ_max_, Δρ_min_ (e Å^−3^)	0.36, −0.44

Computer programs: *CrysAlis PRO* (Rigaku OD, 2018 ▸), *SHELXS* (Sheldrick, 2015*a* ▸), *SHELXL2014* (Sheldrick, 2015*b* ▸), *ORTEP-3 for Windows* (Farrugia, 2012 ▸), *DIAMOND* (Brandenburg, 2006 ▸) and *publCIF* (Westrip, 2010 ▸).

## supplementary crystallographic information

### Crystal data

**Table:**

[Sn(C_6_H_5_)_3_(C_7_H_14_NS_2_)]	*Z* = 2
*M**_r_* = 526.30	*F*(000) = 536
Triclinic, *P*1	*D*_x_ = 1.469 Mg m^−^^3^
*a* = 9.7572 (1) Å	Cu *K*α radiation, λ = 1.54184 Å
*b* = 11.7030 (2) Å	Cell parameters from 24601 reflections
*c* = 11.7602 (2) Å	θ = 3.9–76.3°
α = 74.419 (1)°	µ = 10.25 mm^−^^1^
β = 80.114 (1)°	*T* = 100 K
γ = 67.285 (2)°	Prism, colourless
*V* = 1189.71 (4) Å^3^	0.12 × 0.11 × 0.08 mm

### Data collection

**Table:**

Rigaku XtaLAB Synergy Dualflex AtlasS2 diffractometer	4231 reflections with *I* > 2σ(*I*)
Detector resolution: 5.2558 pixels mm^-1^	*R*_int_ = 0.022
ω scans	θ_max_ = 67.1°, θ_min_ = 3.9°
Absorption correction: gaussian (CrysAlis PRO; Rigaku OD, 2018)	*h* = −11→11
*T*_min_ = 0.840, *T*_max_ = 1.000	*k* = −13→13
28219 measured reflections	*l* = −13→14
4248 independent reflections	

### Refinement

**Table:**

Refinement on *F*^2^	Primary atom site location: structure-invariant direct methods
Least-squares matrix: full	Secondary atom site location: difference Fourier map
*R*[*F*^2^ > 2σ(*F*^2^)] = 0.015	Hydrogen site location: inferred from neighbouring sites
*wR*(*F*^2^) = 0.040	H-atom parameters constrained
*S* = 1.00	*w* = 1/[σ^2^(*F*_o_^2^) + (0.026*P*)^2^ + 0.5977*P*] where *P* = (*F*_o_^2^ + 2*F*_c_^2^)/3
4248 reflections	(Δ/σ)_max_ = 0.001
266 parameters	Δρ_max_ = 0.36 e Å^−^^3^
0 restraints	Δρ_min_ = −0.44 e Å^−^^3^

### Special details

**Table:**

Geometry. All esds (except the esd in the dihedral angle between two l.s. planes) are estimated using the full covariance matrix. The cell esds are taken into account individually in the estimation of esds in distances, angles and torsion angles; correlations between esds in cell parameters are only used when they are defined by crystal symmetry. An approximate (isotropic) treatment of cell esds is used for estimating esds involving l.s. planes.

### Fractional atomic coordinates and isotropic or equivalent isotropic displacement parameters (Å^2^)

**Table:**

	*x*	*y*	*z*	*U*_iso_*/*U*_eq_	
Sn	0.39008 (2)	0.31848 (2)	0.21713 (2)	0.01326 (4)	
S1	0.19166 (4)	0.50402 (3)	0.11108 (3)	0.01639 (8)	
S2	0.17439 (4)	0.47895 (4)	0.36811 (3)	0.01880 (8)	
N1	−0.00996 (14)	0.67737 (12)	0.22420 (12)	0.0176 (3)	
C1	0.10482 (15)	0.56682 (14)	0.23615 (14)	0.0157 (3)	
C2	−0.08409 (18)	0.72965 (17)	0.33124 (16)	0.0259 (4)	
H2	−0.0355	0.6655	0.4017	0.031*	
C3	−0.24831 (19)	0.74782 (18)	0.34878 (17)	0.0301 (4)	
H3A	−0.3009	0.8147	0.2840	0.045*	
H3B	−0.2902	0.7723	0.4243	0.045*	
H3C	−0.2600	0.6682	0.3493	0.045*	
C4	−0.0574 (2)	0.8507 (2)	0.3257 (2)	0.0454 (5)	
H4A	0.0498	0.8342	0.3141	0.068*	
H4B	−0.0976	0.8788	0.3998	0.068*	
H4C	−0.1073	0.9170	0.2594	0.068*	
C5	−0.07259 (17)	0.75964 (14)	0.11003 (14)	0.0196 (3)	
H5	−0.1546	0.8354	0.1323	0.023*	
C6	−0.1481 (2)	0.69886 (17)	0.05404 (17)	0.0273 (4)	
H6A	−0.2025	0.7616	−0.0122	0.041*	
H6B	−0.2180	0.6691	0.1132	0.041*	
H6C	−0.0727	0.6267	0.0250	0.041*	
C7	0.03571 (19)	0.81240 (16)	0.02586 (16)	0.0278 (4)	
H7A	0.1147	0.7437	−0.0056	0.042*	
H7B	0.0798	0.8497	0.0683	0.042*	
H7C	−0.0173	0.8779	−0.0396	0.042*	
C11	0.55885 (16)	0.34926 (13)	0.28704 (13)	0.0147 (3)	
C12	0.68313 (16)	0.35637 (15)	0.21077 (14)	0.0191 (3)	
H12	0.6918	0.3435	0.1331	0.023*	
C13	0.79426 (17)	0.38199 (16)	0.24707 (15)	0.0228 (3)	
H13	0.8761	0.3902	0.1931	0.027*	
C14	0.78624 (17)	0.39561 (15)	0.36188 (16)	0.0220 (3)	
H14	0.8633	0.4115	0.3872	0.026*	
C15	0.66454 (17)	0.38591 (14)	0.43962 (15)	0.0195 (3)	
H15	0.6594	0.3934	0.5188	0.023*	
C16	0.55062 (16)	0.36527 (14)	0.40185 (14)	0.0167 (3)	
H16	0.4661	0.3620	0.4546	0.020*	
C21	0.31244 (16)	0.16861 (14)	0.31013 (13)	0.0161 (3)	
C22	0.40747 (18)	0.05244 (15)	0.37020 (15)	0.0215 (3)	
H22	0.5088	0.0403	0.3739	0.026*	
C23	0.3558 (2)	−0.04562 (16)	0.42466 (16)	0.0256 (4)	
H23	0.4216	−0.1239	0.4661	0.031*	
C24	0.20910 (19)	−0.03021 (16)	0.41896 (15)	0.0227 (3)	
H24	0.1740	−0.0975	0.4565	0.027*	
C25	0.11378 (18)	0.08408 (17)	0.35814 (16)	0.0255 (4)	
H25	0.0132	0.0950	0.3529	0.031*	
C26	0.16529 (18)	0.18255 (16)	0.30497 (15)	0.0234 (3)	
H26	0.0989	0.2610	0.2643	0.028*	
C31	0.50078 (15)	0.25703 (14)	0.05537 (14)	0.0162 (3)	
C32	0.55917 (18)	0.12811 (15)	0.05353 (16)	0.0229 (3)	
H32	0.5551	0.0668	0.1246	0.027*	
C33	0.62313 (19)	0.08811 (16)	−0.05083 (17)	0.0282 (4)	
H33	0.6622	0.0000	−0.0505	0.034*	
C34	0.63013 (19)	0.17592 (18)	−0.15492 (16)	0.0272 (4)	
H34	0.6732	0.1483	−0.2262	0.033*	
C35	0.57407 (19)	0.30455 (17)	−0.15524 (15)	0.0249 (3)	
H35	0.5790	0.3653	−0.2265	0.030*	
C36	0.51064 (17)	0.34382 (15)	−0.05060 (14)	0.0197 (3)	
H36	0.4731	0.4319	−0.0512	0.024*	

### Atomic displacement parameters (Å^2^)

**Table:**

	*U*^11^	*U*^22^	*U*^33^	*U*^12^	*U*^13^	*U*^23^
Sn	0.01301 (6)	0.01352 (6)	0.01339 (6)	−0.00463 (4)	−0.00068 (4)	−0.00360 (4)
S1	0.01698 (17)	0.01653 (17)	0.01422 (18)	−0.00254 (13)	−0.00113 (13)	−0.00638 (13)
S2	0.01615 (17)	0.02318 (18)	0.01490 (18)	−0.00338 (14)	−0.00031 (13)	−0.00680 (14)
N1	0.0147 (6)	0.0194 (6)	0.0190 (7)	−0.0035 (5)	−0.0015 (5)	−0.0083 (5)
C1	0.0122 (6)	0.0189 (7)	0.0187 (8)	−0.0070 (6)	0.0006 (5)	−0.0076 (6)
C2	0.0201 (8)	0.0305 (9)	0.0226 (9)	0.0009 (7)	0.0008 (6)	−0.0153 (7)
C3	0.0253 (9)	0.0306 (9)	0.0286 (10)	−0.0063 (7)	0.0080 (7)	−0.0087 (7)
C4	0.0423 (11)	0.0616 (14)	0.0523 (14)	−0.0251 (10)	0.0112 (10)	−0.0445 (12)
C5	0.0184 (7)	0.0170 (7)	0.0221 (8)	−0.0032 (6)	−0.0043 (6)	−0.0053 (6)
C6	0.0278 (9)	0.0265 (9)	0.0297 (10)	−0.0086 (7)	−0.0116 (7)	−0.0057 (7)
C7	0.0280 (9)	0.0232 (8)	0.0295 (10)	−0.0081 (7)	0.0028 (7)	−0.0061 (7)
C11	0.0136 (6)	0.0116 (6)	0.0173 (8)	−0.0025 (5)	−0.0032 (5)	−0.0026 (5)
C12	0.0153 (7)	0.0211 (7)	0.0178 (8)	−0.0027 (6)	−0.0015 (6)	−0.0048 (6)
C13	0.0134 (7)	0.0265 (8)	0.0256 (9)	−0.0058 (6)	0.0005 (6)	−0.0045 (7)
C14	0.0154 (7)	0.0208 (8)	0.0312 (9)	−0.0049 (6)	−0.0071 (6)	−0.0068 (7)
C15	0.0217 (7)	0.0157 (7)	0.0200 (8)	−0.0025 (6)	−0.0053 (6)	−0.0065 (6)
C16	0.0159 (7)	0.0142 (7)	0.0178 (8)	−0.0031 (5)	−0.0009 (6)	−0.0038 (6)
C21	0.0192 (7)	0.0169 (7)	0.0139 (7)	−0.0078 (6)	0.0014 (6)	−0.0058 (6)
C22	0.0210 (8)	0.0211 (8)	0.0230 (8)	−0.0084 (6)	−0.0041 (6)	−0.0029 (6)
C23	0.0315 (9)	0.0184 (8)	0.0249 (9)	−0.0081 (7)	−0.0041 (7)	−0.0019 (7)
C24	0.0314 (9)	0.0233 (8)	0.0192 (8)	−0.0168 (7)	0.0075 (6)	−0.0094 (6)
C25	0.0204 (8)	0.0312 (9)	0.0291 (9)	−0.0140 (7)	0.0047 (7)	−0.0104 (7)
C26	0.0182 (8)	0.0229 (8)	0.0264 (9)	−0.0060 (6)	−0.0006 (6)	−0.0036 (7)
C31	0.0131 (7)	0.0189 (7)	0.0179 (8)	−0.0054 (6)	−0.0003 (5)	−0.0071 (6)
C32	0.0225 (8)	0.0194 (8)	0.0260 (9)	−0.0062 (6)	0.0008 (6)	−0.0074 (7)
C33	0.0265 (8)	0.0225 (8)	0.0389 (10)	−0.0074 (7)	0.0034 (7)	−0.0180 (8)
C34	0.0241 (8)	0.0378 (10)	0.0267 (9)	−0.0127 (7)	0.0062 (7)	−0.0212 (8)
C35	0.0270 (8)	0.0312 (9)	0.0189 (8)	−0.0134 (7)	0.0027 (6)	−0.0079 (7)
C36	0.0201 (7)	0.0201 (8)	0.0201 (8)	−0.0071 (6)	0.0001 (6)	−0.0078 (6)

### Geometric parameters (Å, º)

**Table:**

Sn—S1	2.4792 (4)	C12—H12	0.9500
Sn—S2	2.9264 (4)	C13—C14	1.387 (3)
Sn—C11	2.1446 (14)	C13—H13	0.9500
Sn—C21	2.1349 (15)	C14—C15	1.391 (2)
Sn—C31	2.1754 (15)	C14—H14	0.9500
C1—S1	1.7587 (15)	C15—C16	1.388 (2)
C1—S2	1.7006 (16)	C15—H15	0.9500
C1—N1	1.336 (2)	C16—H16	0.9500
C5—N1	1.495 (2)	C21—C26	1.391 (2)
C2—N1	1.497 (2)	C21—C22	1.394 (2)
C2—C3	1.517 (2)	C22—C23	1.387 (2)
C2—C4	1.519 (3)	C22—H22	0.9500
C2—H2	1.0000	C23—C24	1.383 (2)
C3—H3A	0.9800	C23—H23	0.9500
C3—H3B	0.9800	C24—C25	1.385 (3)
C3—H3C	0.9800	C24—H24	0.9500
C4—H4A	0.9800	C25—C26	1.387 (2)
C4—H4B	0.9800	C25—H25	0.9500
C4—H4C	0.9800	C26—H26	0.9500
C5—C7	1.515 (2)	C31—C32	1.397 (2)
C5—C6	1.519 (2)	C31—C36	1.396 (2)
C5—H5	1.0000	C32—C33	1.391 (3)
C6—H6A	0.9800	C32—H32	0.9500
C6—H6B	0.9800	C33—C34	1.382 (3)
C6—H6C	0.9800	C33—H33	0.9500
C7—H7A	0.9800	C34—C35	1.388 (3)
C7—H7B	0.9800	C34—H34	0.9500
C7—H7C	0.9800	C35—C36	1.390 (2)
C11—C12	1.395 (2)	C35—H35	0.9500
C11—C16	1.397 (2)	C36—H36	0.9500
C12—C13	1.388 (2)		
			
C21—Sn—C11	119.87 (6)	C12—C11—C16	118.47 (14)
C21—Sn—C31	102.73 (6)	C12—C11—Sn	117.04 (11)
C11—Sn—C31	103.38 (5)	C16—C11—Sn	124.47 (11)
C21—Sn—S1	112.27 (4)	C13—C12—C11	120.79 (15)
C11—Sn—S1	119.12 (4)	C13—C12—H12	119.6
C31—Sn—S1	93.24 (4)	C11—C12—H12	119.6
C21—Sn—S2	88.10 (4)	C14—C13—C12	120.24 (15)
C11—Sn—S2	86.68 (4)	C14—C13—H13	119.9
C31—Sn—S2	158.41 (4)	C12—C13—H13	119.9
S1—Sn—S2	65.260 (11)	C13—C14—C15	119.49 (15)
C1—S1—Sn	95.87 (5)	C13—C14—H14	120.3
C1—S2—Sn	82.36 (5)	C15—C14—H14	120.3
C1—N1—C5	125.74 (13)	C14—C15—C16	120.21 (15)
C1—N1—C2	119.61 (13)	C14—C15—H15	119.9
C5—N1—C2	114.62 (12)	C16—C15—H15	119.9
N1—C1—S2	123.73 (12)	C15—C16—C11	120.71 (14)
N1—C1—S1	119.94 (12)	C15—C16—H16	119.6
S2—C1—S1	116.33 (8)	C11—C16—H16	119.6
N1—C2—C3	111.83 (14)	C26—C21—C22	118.14 (14)
N1—C2—C4	110.44 (15)	C26—C21—Sn	119.77 (11)
C3—C2—C4	112.51 (15)	C22—C21—Sn	121.95 (11)
N1—C2—H2	107.3	C23—C22—C21	120.74 (15)
C3—C2—H2	107.3	C23—C22—H22	119.6
C4—C2—H2	107.3	C21—C22—H22	119.6
C2—C3—H3A	109.5	C24—C23—C22	120.43 (15)
C2—C3—H3B	109.5	C24—C23—H23	119.8
H3A—C3—H3B	109.5	C22—C23—H23	119.8
C2—C3—H3C	109.5	C23—C24—C25	119.52 (15)
H3A—C3—H3C	109.5	C23—C24—H24	120.2
H3B—C3—H3C	109.5	C25—C24—H24	120.2
C2—C4—H4A	109.5	C24—C25—C26	119.94 (15)
C2—C4—H4B	109.5	C24—C25—H25	120.0
H4A—C4—H4B	109.5	C26—C25—H25	120.0
C2—C4—H4C	109.5	C21—C26—C25	121.22 (15)
H4A—C4—H4C	109.5	C21—C26—H26	119.4
H4B—C4—H4C	109.5	C25—C26—H26	119.4
N1—C5—C7	113.59 (13)	C32—C31—C36	117.70 (14)
N1—C5—C6	112.50 (13)	C32—C31—Sn	120.43 (12)
C7—C5—C6	114.30 (15)	C36—C31—Sn	121.81 (11)
N1—C5—H5	105.1	C33—C32—C31	120.96 (16)
C7—C5—H5	105.1	C33—C32—H32	119.5
C6—C5—H5	105.1	C31—C32—H32	119.5
C5—C6—H6A	109.5	C34—C33—C32	120.27 (16)
C5—C6—H6B	109.5	C34—C33—H33	119.9
H6A—C6—H6B	109.5	C32—C33—H33	119.9
C5—C6—H6C	109.5	C33—C34—C35	119.89 (16)
H6A—C6—H6C	109.5	C33—C34—H34	120.1
H6B—C6—H6C	109.5	C35—C34—H34	120.1
C5—C7—H7A	109.5	C36—C35—C34	119.53 (16)
C5—C7—H7B	109.5	C36—C35—H35	120.2
H7A—C7—H7B	109.5	C34—C35—H35	120.2
C5—C7—H7C	109.5	C35—C36—C31	121.64 (15)
H7A—C7—H7C	109.5	C35—C36—H36	119.2
H7B—C7—H7C	109.5	C31—C36—H36	119.2
			
C5—N1—C1—S2	178.33 (11)	C13—C14—C15—C16	−1.3 (2)
C2—N1—C1—S2	0.0 (2)	C14—C15—C16—C11	2.4 (2)
C5—N1—C1—S1	−2.2 (2)	C12—C11—C16—C15	−0.9 (2)
C2—N1—C1—S1	179.49 (11)	Sn—C11—C16—C15	−179.42 (11)
Sn—S2—C1—N1	−176.75 (13)	C26—C21—C22—C23	−0.9 (2)
Sn—S2—C1—S1	3.78 (7)	Sn—C21—C22—C23	−176.59 (13)
Sn—S1—C1—N1	176.06 (11)	C21—C22—C23—C24	0.8 (3)
Sn—S1—C1—S2	−4.44 (8)	C22—C23—C24—C25	0.1 (3)
C1—N1—C2—C3	−120.82 (16)	C23—C24—C25—C26	−0.9 (3)
C5—N1—C2—C3	60.70 (19)	C22—C21—C26—C25	0.1 (2)
C1—N1—C2—C4	113.06 (18)	Sn—C21—C26—C25	175.91 (13)
C5—N1—C2—C4	−65.42 (18)	C24—C25—C26—C21	0.8 (3)
C1—N1—C5—C7	−64.0 (2)	C36—C31—C32—C33	−0.8 (2)
C2—N1—C5—C7	114.38 (15)	Sn—C31—C32—C33	176.56 (12)
C1—N1—C5—C6	67.84 (19)	C31—C32—C33—C34	0.1 (3)
C2—N1—C5—C6	−113.79 (15)	C32—C33—C34—C35	0.5 (3)
C16—C11—C12—C13	−1.6 (2)	C33—C34—C35—C36	−0.3 (3)
Sn—C11—C12—C13	176.97 (12)	C34—C35—C36—C31	−0.4 (2)
C11—C12—C13—C14	2.7 (2)	C32—C31—C36—C35	1.0 (2)
C12—C13—C14—C15	−1.3 (2)	Sn—C31—C36—C35	−176.35 (12)

### Hydrogen-bond geometry (Å, º)

**Table:**

*D*—H···*A*	*D*—H	H···*A*	*D*···*A*	*D*—H···*A*
C34—H34···C24^i^	0.95	2.82	3.757 (3)	171

Symmetry code: (i) −*x*+1, −*y*, −*z*.
